# Gene Therapy in Hemophilia: From Hype to Hope

**DOI:** 10.1097/HS9.0000000000000037

**Published:** 2018-04-16

**Authors:** Roger Schutgens

**Affiliations:** University Medical Center and University Utrecht, Utrecht, The Netherlands

Hemophilia is an X-linked disorder caused by mutations in the factor VIII (*FVIII*) or factor IX (*FIX*) gene, leading to an increased bleeding tendency. Since the 1970s, routine administration of clotting factor concentrates changed hemophilia from a life-threatening and incapacitating disease to a chronic disorder with 1 to 2 bleeds each year, good quality of life and equal life expectancy to the general population. Currently, the use of extended half-life FVIII and FIX products have made their way into clinical practice, leading to increased trough levels or less frequent infusions. Although clotting factor concentrates have been improved in terms of manufacturing processes, safety, and bioengineering, the main treatment strategy of supplementing the missing clotting factor has been the same for over 50 years.

Recently, new treatment modalities have emerged in clinical trials, using innovative means to mimic the native FVIII function (emicizumab) or to downregulate natural anticoagulants (fitusiran or anti-Tissue Factor Pathway Inhibitor antibodies). Although the first efficacy results are promising, several safety issues (especially thrombotic events) remain to be resolved. Meanwhile, the long-lasting search for gene therapy is finally coming to a clinical stage of development.

**Figure 1:**
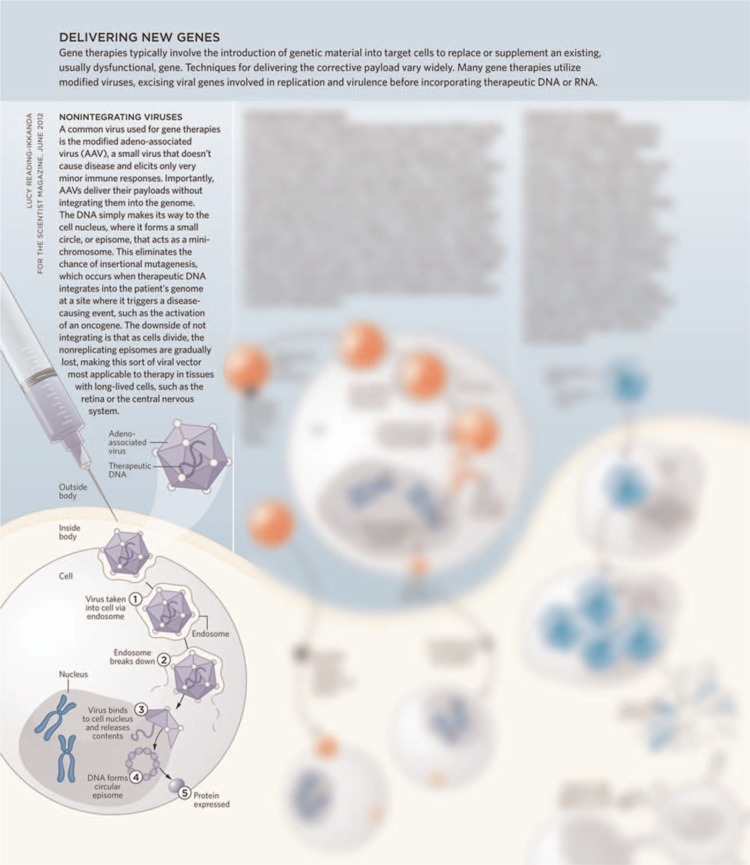
**From: The Scientist, June 2012 Issue, by Jef Akst.** (https://www.the-scientist.com/?articles.view/articleNo/32141/title/Targeting-DNA/).

In December 2017, 3 gene therapy trials were published^1–3^ (Table 1). All 3 trials used adeno-associated viral (AAV) vectors for gene delivery. The AAV vectors are based on a nonpathogenic parvovirus that is not able to replicate itself. The viral code sequences are replaced by a gene expression cassette of interest.^4^ AAV vectors are nonintegrating; transgene DNA is stabilized predominantly episomally. This has the advantage of a reduced risk of insertional mutagenesis, but the disadvantage of being dependent on long-lived postmitotic cells for long-term expression (Fig. 1).

**Table 1:**
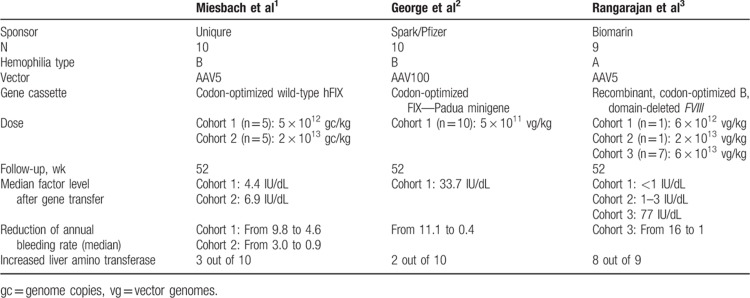
Overview of recent gene therapy trials in severe and moderate hemophilia

All trials used a liver-specific promotor. Safety was the primary endpoint in the trials, with emphasis on liver toxicity by measuring alanine aminotransferase (ALT) levels. In those patients with increased ALT levels, a course of prednisone 60 mg was given. In all of these patients, ALT levels dropped within normal range without loss of response. ALT elevations are thought to have an immunological basis, but no cytotoxic T-cell response was found against the capsid or transduced hepatocytes in either the Rangarajan or the Miesbach study. No other clinically significant safety issues were observed in the trials.

Efficacy was evaluated by measuring factor levels after gene transfer. From the hemophilia B trials, it is clear that prolonged expression of FIX levels was established after a single infusion of the vector. In the Uniqure trial, FIX levels were slightly higher in the higher-dose cohort. In the SPARK trial, the *FIX*-Padua gene was used. This is a naturally occurring gain-of-function mutation in the *FIX* gene associated with hyperfunctional activity of the protein with FIX levels up to 776 IU/dL. With this *FIX*-Padua gene cassette, a median level of expression of 33.7 IU/dL was reached. In the hemophilia A trial, the low and intermediate dose did not result in transformation to a mild phenotype, but the patients treated with the high dose all reached FVIII levels >5 IU/dL after 16 weeks. After 20 weeks, in 6 out of 7 patients FVIII levels >50 IU/dL were noted. Importantly, annual bleeding episodes reduced dramatically after gene transfer.

The first report on long-lasting protein expression after gene therapy in hemophilia using an AAV vector dated from 2011.^5^ Emerging genome editing technologies now further advance the scope and efficacy of gene therapy approaches.^4^ These include improved vector cassette design and advanced production facilities in a mammalian expression system or by transfection of insect cells with baculovirus.^6^ The recent developments in this field clearly indicate that this therapy is now being developed on a commercial therapeutic basis.

In conclusion, our hope for sustainable clotting factor levels after a single infusion has become reality. The current gene transfer trials appear to be safe and efficacious. A prosperous future lies ahead.
